# Production of Fungal Glucoamylase for Glucose Production from Food Waste

**DOI:** 10.3390/biom3030651

**Published:** 2013-09-18

**Authors:** Wan Chi Lam, Daniel Pleissner, Carol Sze Ki Lin

**Affiliations:** School of Energy and Environment, City University of Hong Kong, Tat Chee Avenue, Kowloon, Hong Kong, China; E-Mails: wanclam@cityu.edu.hk (W.C.L.); dpleissn@cityu.edu.hk (D.P.); carollin@cityu.edu.hk (C.S.K.L)

**Keywords:** glucoamylase production, *Aspergillus awamori*, food waste hydrolysis, glucose production

## Abstract

The feasibility of using pastry waste as resource for glucoamylase (GA) production via solid state fermentation (SSF) was studied. The crude GA extract obtained was used for glucose production from mixed food waste. Our results showed that pastry waste could be used as a sole substrate for GA production. A maximal GA activity of 76.1 ± 6.1 U/mL was obtained at Day 10. The optimal pH and reaction temperature for the crude GA extract for hydrolysis were pH 5.5 and 55 °C, respectively. Under this condition, the half-life of the GA extract was 315.0 minutes with a deactivation constant (k_d_) 2.20 × 10^−3^ minutes^−1^. The application of the crude GA extract for mixed food waste hydrolysis and glucose production was successfully demonstrated. Approximately 53 g glucose was recovered from 100 g of mixed food waste in 1 h under the optimal digestion conditions, highlighting the potential of this approach as an alternative strategy for waste management and sustainable production of glucose applicable as carbon source in many biotechnological processes.

## 1. Introduction

Food waste is a serious global problem, especially in many developed countries. In Hong Kong, over 3500 tons of food wastes are generated every day [1]. Currently, landfilling and incineration are the major practices for managing these wastes in many countries. These practices, however, may cause severe environmental pollutions and adds burden to the economy. Due to its high contents of carbohydrates and proteins, food wastes may serve as feedstock in biorefineries for production of fungal enzymes, e.g., glucoamylase (GA) and offers an innovative approach to waste management.

GA is a family of amylolytic enzymes that catalyze the cleavage of α-(1,4) glycosidic bonds in starch and release glucose as end product [2,3]. Glucose is the principle carbon source in many biotechnological processes and of great importance for fermentative chemicals and fuel production such as succinic acid, bio-plastic and ethanol. Starch is usually the major component of mixed food waste from restaurants [4,5,6], application of GA for food waste hydrolysis to recover glucose from starch, therefore, may not only offer a solution for managing food waste but also help to save precious resources.

*Aspergillus awamori* is a known secretor of GA with beneficial properties for industrial bioprocesses such as high productivity and enzyme activity at high temperatures [3,7]. Therefore, *Aspergillus awamori* is employed in this study for GA production through solid state fermentation (SSF). SSF is a fermentation process conducted in the absence of free water, thus it is desirable for industrial enzymes production since the enzymes produced at the end are not diluted by the amount water added in comparison to submerge fermentation. Consequently, the enzymes produced are at a much higher concentration. Pastry waste collected from local Starbucks contains 44.6% of starch and 7% of protein [8]. Starch has been shown as an inducer for GA synthesis by some *Aspergillus* producers [9,10,11]; thus, the significant amount of starch in pastry waste could be desirable for GA production. The smaller amount of protein, 7%, on the other hand could provide a source of nitrogen to promote the fungal growth and facilitate GA production. Therefore, pastry waste was selected in this study for GA production. The crude GA extract obtained without further purification was characterized in terms of optimal pH and reaction temperature, as well as thermo-stability. Additionally, application of the crude GA extract was studied for hydrolysis of mixed food waste collected from a local restaurant to produce high glucose solution.

## 2. Results and Discussion

### 2.1. Glucoamylase Production from Pastry Waste

**Figure 1:**
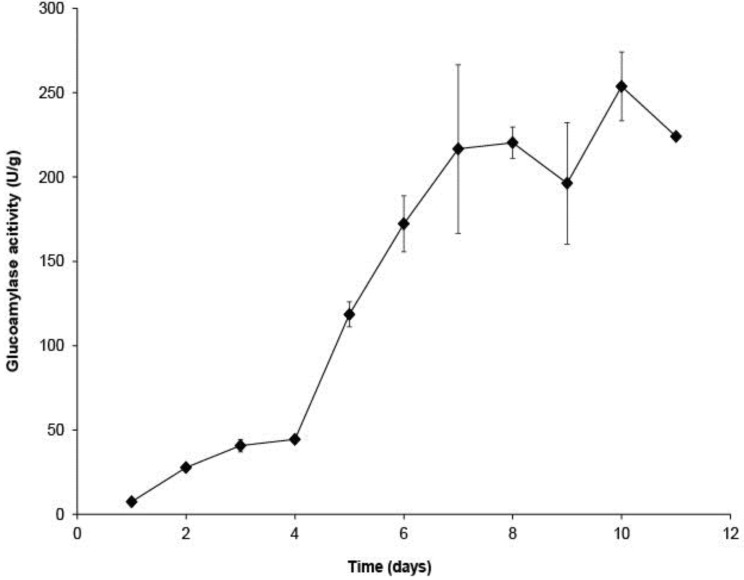
Glucoamylase (GA) production from pastry waste with *Aspergillus awamori* during solid state fermentation(SSF) for 11 days at 30°C. Experiments were duplicated. The mean values are plotted and the standard errors are reported.

To demonstrate the feasibility of pastry waste as the sole substrate for GA production, SSF was conducted with *Aspergillus awamori* without addition of any other nitrogen or carbon sources. GA production from pastry waste over time is shown in Figure 1. Production of GA reached maximal activity at Day 10. Approximately, 253.7 ± 20.4 U of GA was produced from one gram of pastry waste on dry basis (d.b.); the GA activity of the crude GA extract was 76.1 ± 6.1 U/mL. The result demonstrates that pastry waste can be used solely for GA production.

GA production with different wastes as substrates have been studied and the results are summarized in Table 1. In some studies, nitrogen in form of ammonium, urea and yeast extract was supplemented to substrates to facilitate fungal growth and GA production [4,12,13,14]. In contrast, nitrogen supplement was not involved in this study, but the GA activity of enzyme extract appears higher than in earlier studies with nitrogen supplement most likely due to a good balance of carbon (C) to nitrogen (N) and phosphorus (P) ratios. The data suggests that pastry waste is a promising substrate for GA production.

**Table 1:** GA production and yields from different studied substrates with or without nitrogen supplement through solid state fermentation.

Substrate	Crude GA concentration (U/mL)	Yield (U/g)	Fungus	Nitrogen supplement	References
Rice powder	N/A	71.3 ± 2.34 ^a^	*Aspergillus niger*	+	[12]
Wheat bran	N/A	110 ± 1.32 ^a^	*Aspergillus niger*	+	[12]
Mixed food waste	137	N/A	*Aspergillus niger*	+	[13]
Cowpea waste	970	N/A	*Aspergillus oryzae*	-	[14]
Wheat bran	4.4	48	*Aspergillus awamori*	-	[15]
Wheat pieces	3.32	81.3	*Aspergillus awamori*	-	[16]
Waste bread	3.94	78.4	*Aspergillus awamori*	-	[16]
Waste bread	N/A	114	*Aspergillus awamori*	-	[17]
Pastry waste	76.1 ± 6.1 ^a^	253.7 ± 20.4 ^a^	*Aspergillus awamori*	-	This study

^a^ Values indicate means ± standard errors.

### 2.2. Characterization of Optimal Reaction Temperature and pH of the Crude Glucoamylase Extract

**Figure 2:**
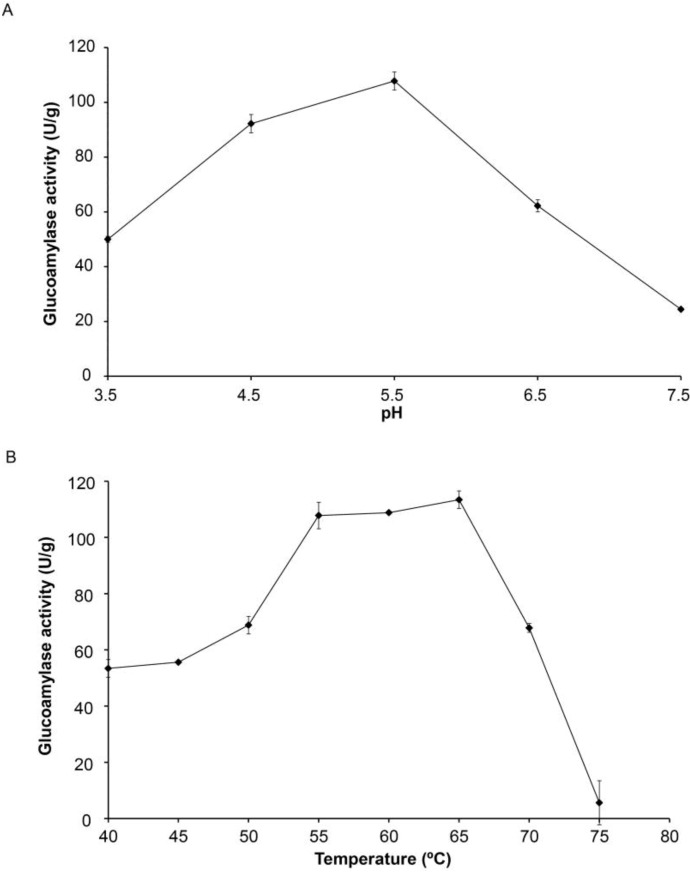
Effect of (**A**) pH at 55°C and (**B**) temperature at pH 5.5 on crude GA extract activity. Experiments were duplicated. The mean values are plotted and the standard errors are reported.

Optimal pH and reaction temperature of the crude GA extract were determined. The results are shown in Figure 2. In order to determine the optimal reaction pH of the crude GA extract, assays were carried out at various pHs from 3.5 to 7.5 and the results are indicated in Figure 2A. The maximal enzyme activity was obtained at pH 5.5, indicating it was the optimal pH for starch hydrolysis. Fungal GAs from *Aspergillus* strains are usually active at acidic pH, their enzyme activities vary from pH 3.5 to 7 depending on the strains and amino acid sequences (isoform) [3]. Similarly, GA assay was conducted at different temperatures as indicated in Figure 2B from 40–75 °C with 5 °C increment at pH 5.5 in order to determine the optimal reaction temperature for the crude GA extract and assuming that the pH factor is independent of the temperature factor. A significant increase in enzyme activity was observed as temperature increases from 40 to 55 °C. Maximal GA activity was observed from 55 to 65 °C suggesting the range for optimal reaction temperature of the crude GA extract and in accordance with optimal reaction temperatures in the range of 50 to 60 °C usually found for GAs from *Aspergillus* [3]. Further increase in reaction temperature greatly reduced the enzyme activity most likely due to enzyme denaturation.

### 2.3. Thermo-Stability of the Crude Glucoamylase Extract at Optimal Reaction Temperatures

**Figure 3:**
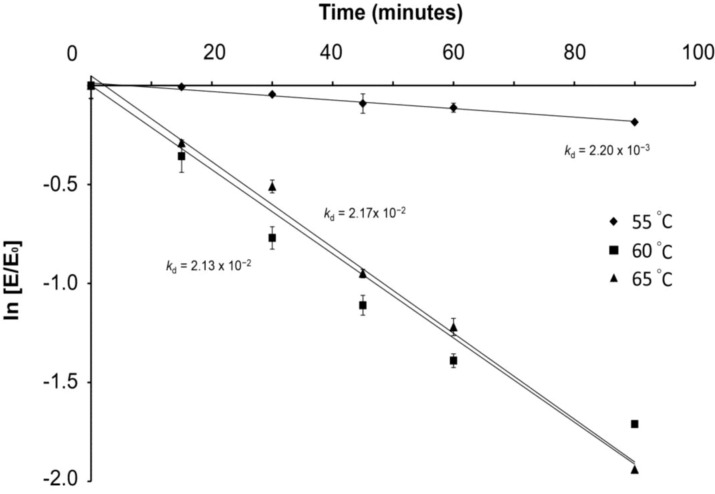
Thermal deactivation of crude GA extract at (◆) 55°C, (■) 60°C and (▲) 65°C over 90 min at pH 5.5. Experiments were duplicated. The mean values are plotted and the standard errors were reported.

Since high GA activity was observed at 55, 60 and 65 °C, thermo-stability of the crude GA extract at these temperatures was further investigated in order to determine the optimal digestion temperature for the subsequent food waste hydrolysis experiment. The residual enzyme activity after heated at 55, 60 and 65 °C for over 90 min is shown in Figure 3. The rate constant (k_d_, minutes^−1^) for the first-order thermal deactivation was determined from the slope of the deactivation time course as shown in Figure 3 using Equation (1) [18], where E_t_ is the residual GA activity after heat treatment for time t. E_0_ is the initial enzyme activity before heat treatment. The half-life of thermal deactivation (t_1/2_) was determined according to Equation (2) [19]. The thermal deactivation of the crude GA extract exhibited a linear relationship showing that it followed first-order kinetics as reported [20]. The thermal deactivation rate constant k_d_ (minutes^−1^) of the crude GA extract at 55 °C was found approximately 10 times slower than the rates at 60 and 65 °C, suggesting the crude GA is more thermo-stable at 55 °C. The k_d_ of the crude GA extract at 55 °C was 2.20 × 10^−3^ in comparison with 2.13 × 10^−2^ and 2.17 × 10^−2^ at 60 and 65 °C, respectively. The half-life (t_1/2_) of the enzyme extract at 55 °C was 315 min in comparison with 32.5 and 31.9 min for 60 and 65 °C, respectively (Table 2). Since the enzymatic activity of the crude GA extract at 55 °C was close to the activity at 60 and 65 °C, but more stable, it was adopted as the optimal digestion temperature for the subsequent food hydrolysis experiment.


ln (E_t_/E_0_) = −k_d_t (1)

t_1/2_ = ln(2)/k_d_ (2)

**Table 2:** Deactivation constant (k_d_) and half-lives (t_1/2_) of the crude GA extract at 55, 60 and 65°C at pH 5.5.

Temperature (°C)	k_d_ (minutes^−1^)	t_1/2 _(minutes)
55	2.20 × 10^−3^	315.0
60	2.13 × 10^−2^	32.5
65	2.17× 10^−2^	31.9

### 2.4. Application of Crude Glucoamylase Extract on Mixed Food Waste Hydrolysis for Glucose Production

**Figure 4:**
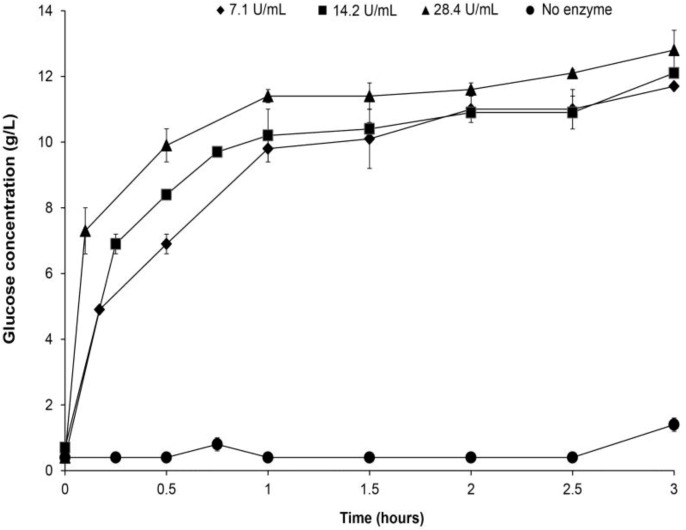
Hydrolysis of mixed food waste for glucose production in the presence of crude GA extract with (●) no enzyme, (◆) 7.1 U/mL, (■) 14.2 U/mL and (▲) 28.4 U/mL at pH 5.5 and 55°C for 3 h.Experiments were duplicated. The mean values are plotted and the standard errors are reported.

In the reality, mixed food waste is rich in salt [21] that may inhibit the enzymatic hydrolysis. To verify if the crude GA extract produced was applicable to food waste digestion for glucose production, it was used to hydrolyse the food waste which was collected from a local restaurant, under its optimal digestion conditions (at pH 5.5 and 55 °C). Increasing concentration of enzyme was added to the food waste and the time required for hydrolysis to produce glucose was determined (Figure 4). Significant difference in glucose concentration was only observed for the first hour but not after. In all cases, the food waste hydrolysis by the enzyme extracts was completed in 1 h. At the end of the hydrolysis, approximately 12 g/L glucose was produced and that was corresponding to approximately 53 g glucose produced from 100 g of mixed food waste (d.b.), while no production of glucose occurred in a control without crude GA extract. The amount of glucose produced from 100 g of mixed food waste hydrolysis was consistent with our previous study using a different hydrolysis approach [5].

The two commonly used approaches for cereal-based waste and food waste hydrolysis to produce glucose rich solution, include simultaneous fungus culturing and hydrolysis with the enzymes actively secreted [5,8,16,22,23] or direct addition of enzyme solution to digest the substrate [24]. In the first case, food waste hydrolysis is usually completed after 24 h [5,8,16,22,23]. In this study, the latter approach was adopted. Food waste hydrolysis by the crude GA extract was completed in 1 h under optimal conditions found. Similar experiment has been reported by *Yan et al.* using commercial GA for food waste hydrolysis. In their studies, food waste hydrolysis was completed in 2.5 h when GA to substrate ratio reached 80–140 U/g food waste [24]. When the GA to substrate ratio in the solution is reduced to 7.4 U/g substrate, 24 h was needed for complete substrate hydrolysis [16]. The higher efficiency for food waste hydrolysis (in 1 h) in this study was likely due to the higher initial GA to substrate ratio.

### 2.5. Material Balance for Glucose Production from 1 kg Mixed Food Waste with Crude Glucoamylase Extract

Scheme I shows the material balance of the studied process for crude GA production from pastry waste and glucose recovery from 1 kg mixed food waste (d.b.). All the calculations are provided in the supplementary information. According to our study, hydrolysis of 1 kg mixed food waste (d.b.) could lead to 0.53 kg glucose production. Approximately, 1.4 kg pastry waste (d.b.) is required to produce sufficient amount of crude GA extract for 1 kg mixed food waste hydrolysis. Furthermore, the material balance only presents the theoretical values of the up-scaled process based on our laboratory-scale experimental data. However, upscale study is needed in order to demonstrate the process can be applied at industrial scale.

**Scheme I:**
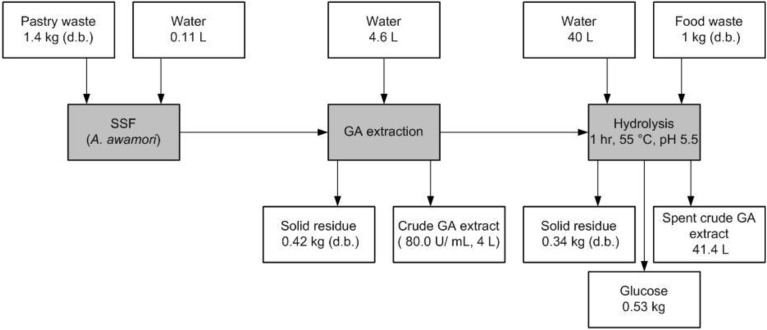
Material balance of the process in this study for processing 1 kg mixed food waste to produce glucose using crude GA extract produced from pastry waste based on the laboratory-scale experimental data.

## 3. Experimental Section

### 3.1. Microorganism

Frozen spores of the fungus *Aspergillus awamori* (ATCC 14331), were used for SSF. Spores were suspended in demineralized water and loaded into conical flasks containing cornmeal agar and incubated at 30 °C for 7 days. Harvesting of fresh spores was carried out using 10% glycerol and the number of spores was counted using a haemocytometer. Fresh spore suspension of 1 mL was diluted to the required concentration with sterile demineralized water and used immediately for SSF.

### 3.2. Food Wastes Preparation

Pastry waste and mixed food waste were obtained from a local Starbucks store and canteen for SSFs and enzymatic hydrolysis experiments, respectively. Once the pastry waste was collected, it was homogenized with a kitchen blender and stored at −20 °C. Pastry waste was autoclaved before subjected to SSF and mixed food waste was lyophilized before enzymatic hydrolysis.

### 3.3. Solid State Fermentation for Glucoamylase Production

Sterilized pastry waste of 20 g was placed in a petri dish and inoculated with 1 mL of fresh spore suspension of *Aspergillus awamori* loaded onto the surface of the substrate. For each g of substrate, 5 × 10^5^ spores were used. The plates were incubated under static condition at 30 °C for 11 days. Whole plate content was withdrawn regularly and analyzed for GA production.

### 3.4. Glucoamylase Extraction

Whole content of the fermented solid in the petri dish was mixed thoroughly in a kitchen blender containing 20 mL demineralized water and the mixture was transferred into a 500 mL Duran bottle. The kitchen blender was rinsed with another portion of 20 mL demineralized water and pooled with the previously obtained mixture. The suspension was then mixed for 30 min with a magnetic stirrer at 30 °C, followed by centrifugation at 22,000 × g for 10 min. The supernatant was collected and filtered with Whitman no. 1 filter paper. The solution obtained refers to crude GA extract.

### 3.5. Food Waste Hydrolysis for Glucose Production

Food waste hydrolysis was conducted by adding increasing amount of crude GA extract to test tubes containing 50 mg dried food waste in 2.2 mL of 0.2 M sodium acetate (pH 5.5). The reaction mixture was incubated at 55 °C for 3 h and mixed by pipetting for every 30 min. Aliquots of samples were withdrawn regularly from the reaction mixture and mixed with 10% (w/v) trichloroacetic acid prior to glucose determination.

### 3.6. Glucoamylase Activity

Activity of the crude GA extract was determined using the method described by Melikoglu *et al.* [17]. Wheat flour solution of 6% (w/v) was used as substrate and it was prepared in sodium acetate (pH 5.5) and gelatinized at 80 °C for 15 min before usage. The assay was conducted by mixing 0.25 mL of 5 times diluted crude GA extract and 0.5 mL of gelatinized wheat flour solution, and incubated at 55 °C. The reaction was terminated after 10 min by adding 0.25 mL 10% (w/v) trichloroacetic acid solution to the reaction mixture. The reaction mixture was centrifuged and the glucose concentration in the supernatant was analyzed using the Analox GL6 glucose analyzer. One unit (U) of GA activity is defined as the amount of enzyme that releases 1 µmol of glucose per minute under assay conditions and is expressed as U/g of dry substrate as described [12].

For determination of optimal reaction pH, the crude GA extract and the gelatinized wheat flour solutions were prepared in sodium acetate buffer from pH 3.5 to 7.5. Optimal reaction temperature determination of the crude GA extract was carried out at temperature range of 40–75 °C with 5 °C increment at optimal pH. Thermo-stability of the crude GA extract was investigated at different temperatures (55–65 °C) at optimal pH. Experiments were performed in duplicate and the standard errors were reported.

## 4. Conclusions

In this work, we have demonstrated the feasibility of pastry waste as feedstock for GA production. High GA yield (253.7 ± 20.4 U/g) of the crude enzyme extract was obtained without addition of nitrogen in comparison to other reported waste substrates, highlighting its potential as a feedstock for GA production in industrial scale. Under the optimal digestion conditions (pH 5.5 and 55 °C), the crude GA extract could hydrolyze mixed food waste in 1 h and generate around 53 g glucose from 100 g of mixed food waste. The work is of great significance as it shows sustainable GA production from food waste for potential municipal food waste treatment and sustainable chemicals production.
